# A Human Health Risk Assessment of Persistent Organic Pollutants in Wild Marine Mussels from the Western Cape Province of South Africa

**DOI:** 10.3390/foods14132226

**Published:** 2025-06-24

**Authors:** Deborah Caitlin Firth, Philip E. Strydom, Lutz Auerswald, Louwrens Christiaan Hoffman

**Affiliations:** 1 Department of Animal Science, Stellenbosch University, Cape Town 7600, South Africa; pestrydom@sun.ac.za (P.E.S.); lutz.auerswald@gmail.com (L.A.); 2 Department of Forestry, Fisheries, and the Environment, Cape Town 8012, South Africa; 3 Centre for Nutrition and Food Sciences, University of Queensland, Brisbane, QLD 4072, Australia

**Keywords:** persistent organic pollutants, mussels, marine, seafood, risk assessment

## Abstract

Persistent Organic Pollutants (POPs) are contaminants that pose potential harm to environments and human consumers. Wild mussels (*Mytilus galloprovincialis*, *Choromytilus meridionalis*, and *Perna perna*) were collected from the coastline of the Western Cape Province of South Africa and analysed for polychlorinated biphenyls (PCBs), organochlorine pesticides (OCPs), and polyaromatic hydrocarbon (PAHs) via gas chromatography tandem mass spectrometry. The results showed eleven PAHs at concentrations ranging from NF to 50.3 ng g^−1^ d.w., five PCBs at concentrations between 4.1 and 18.6 ng g^−1^ d.w., and two OCPs, namely β-hexachlorocyclohexane (NF–7.9 ng g^−1^ d.w.) and chlordane (7.2–14.5 µg g^−1^ d.w.). A Human Health Risk Assessment (HHRA) determined PAH concentrations to pose little health risk to adults and children consuming < 1000 g and 500 g per month (g m^−1^) wild mussel meat, respectively. The HHRA of PCBs found adults and children would experience negative health effects at a consumption rate of 250 g m^−1^. HHRAs determined chlordane concentrations to pose unacceptable health risks for adults and children at all consumption rates (similar results for lindane). To avoid unnecessary POP-related health risks over a lifetime, it is recommended that adults consume < 250 g m^−1^ of wild mussels from the Western Cape Province, and children should avoid consuming mussels. This research demonstrates the legacy of POP contamination along the coastline of the Western Cape Province; more monitoring of these contaminants is imperative to protect marine ecosystems and food chains.

## 1. Introduction

Persistent Organic Pollutants (POPs) are predominantly synthetic compounds produced intentionally or as byproducts of human industry [1]. These chemicals may be released into terrestrial and aquatic environments due to improper waste management, but do not degrade naturally and are therefore known to persist in sediments, waters, plants, and animals [1]. Plants and animals can be exposed to POPs in the environment through oral ingestion, inhalation, and dermal contact [2]. Numerous studies have found elevated concentrations of POPs in marine coastal sediments or species that are correlated with a closer proximity to industrialised or urban spaces, as well as riverine and harbour-based sources of pollution [3,4,5,6]. The lipophilic and hydrophobic properties of POPs result in their accumulation in the fatty tissues of the animals that ingest them, persisting or bioaccumulating up the food chain in the fat of predatory species [7,8,9].

To date, POP exposure in humans has been linked to endocrine disturbances, cardiovascular and reproductive issues, diabetes, obesity, and cancer, along with a wide variety of other, lesser-known effects [2]. In South Africa, POPs have been detected in the blood of some delivering women at concentrations which may create health risks for their infants [10]. The full impact of POP exposure on nature and human populations is hard to estimate because laboratory studies often investigate only the impact of individual POPs or an individual class of POPs [e.g., polychlorinated biphenyls (PCBs), polyaromatic hydrocarbons (PAHs), organochlorine pesticides (OCPs), organophosphate pesticides (OPPs), polybrominated diphenyl ethers (PBDEs), polychlorinated dibenzo-*p*-dioxins (PCDDs or dioxins), polychlorinated dibenzofurans (PCDFs or furans), etc.]. Aside from causing health issues for humans, POPs contribute to the degradation of natural ecosystems, making plants and animals more susceptible to other environmental stressors [2]. These contaminants have been known to impact the integrity of aquatic ecosystems as a whole [11], which can create food safety concerns for humans relying on ocean-harvested foods as sustenance.

Intensive commercial farming in South Africa has resulted in the country being one of the largest importers of pesticides in sub-Saharan Africa [12]. When combined with the growing local automotive industry, the lucrative mining industry, and the diverse chemical industries (fuel and plastic fabrication), there are many potential sources of POP contamination in the country [13]. In September 2004, the South African government entered into the Stockholm Convention on Persistent Organic Pollutants [13]. The Stockholm Convention on POPs was created by the United Nations Environmental Programme (UNEP) with the intention to internationally phase out the use of 12 priority POPs (the “Dirty Dozen”)—aldrin, chlordane, DDT, dieldrin, endrin, heptachlor, hexachlorobenzene, mirex, PCBs, PCDDs, PCDFs, and toxaphene [1,14]. These 12 POPs were found to be transported internationally through oceans and the atmosphere, indicating that international, cooperative efforts were necessary in order to limit or eliminate their prevalence in natural environments [1,14]. Since joining the Stockholm Convention, South Africa has created programmes to remove priority POPs from legal use, as well as to strictly control the ongoing use of DDT in the country for malaria vector control [13].

Despite the wide variety of local contamination sources, many POPs are considered under-researched in the Southern Hemisphere [15]. Marine POP research is scarce within South African publications due to the lack of a large-scale government-funded marine pollution monitoring programme, the high cost of POP analysis, and the scarcity of appropriate analytical equipment available to government and private researchers alike [16]. The only programme that regularly monitors contaminants within local species is the South African Molluscan Shellfish Monitoring and Control Programme (SAMSM&CP), which is run by the Directorate of Sustainable Aquaculture Management [17]. This programme aims to minimise and manage the food safety risks of South African farmed shellfish [the Pacific oyster (*Crassostrea gigas*), the Mediterranean mussel (*Mytilus galloprovincialis*), the local black mussel (*Choromytilus meridionalis*), and the South African abalone (*Haliotis midae*)] that are exported or consumed locally [17]. The focal contaminants in this research are microbiological contaminants in farmed shellfish that require the immediate closure of farms (e.g., *Escheria coli* or harmful algal blooms), and POPs are tested for far less frequently. Other shortcomings of this programme are that it generally does not publish these data and that it also only monitors farmed shellfish, leaving wild shellfish consumers with no protection from potential food-borne contaminants.

While there have been a number of published studies on POP contamination in South African ecosystems in the last 10 years [18], the majority of this research focuses on these compounds in the water and sediments of polluted rivers and estuaries [19,20,21,22,23,24,25,26,27,28,29,30,31]. Research into freshwater contamination by POPs is growing; this is useful to marine pollution researchers, as many of the POPs detected in freshwater ecosystems eventually make their way into the ocean via rivers.

Contaminated seafood is known to be one of the main routes of POP exposure for humans [2,32]. Local research into the POP contamination of marine fish has recently begun to incorporate Human Health Risk Assessments in order to determine the potential risk posed to regular consumers of fish and other seafoods. Research into the risks associated with freshwater fish consumption in South Africa found varying levels of non-cancer and cancer risks to human consumers due to POP contamination in higher-trophic-level freshwater fish from KwaZulu Natal Province [33,34,35], while freshwater fish from the Eastern Cape Province did not pose a risk to regular consumers [36]. Marine fish bought from Kalk Bay harbour in the Western Cape Province of South Africa [snoek (*Thyrsites atun*), bonito (*Sarda orientalis*), panga (*Pachymetopon blochii*), and grey seabream (*Pachymetopon blochii*)] were found to contain emerging pollutants in concentrations that posed both acute and chronic health risks to the fish and human consumers alike [37]. A risk assessment of five indicator herbicides within limpets (*Cymbula granatina*), mussels (*Mytilus galloprovincialis*), and sea urchins (*Parechinus angulosus*) from Camps Bay in the Western Cape Province of South Africa found the potential carcinogenic risk for the regular consumption of coastal invertebrates to be above acceptable limits [38].

There have been a small number of local studies on POP contamination in mussels. These studies tend to focus on one or two contaminant classes at a time, e.g., PCBs [39,40] or PAHs and PCBs [41], and almost all the research has been conducted within harbours or enclosed bays [39,42,43]. The small amount of available research on POPs in local mussels has found them to be ubiquitous along the South African coastline, though contamination levels are typically lower than those detected in mussels from more industrialised nations [4,6]. Gaps in local research therefore include a lack of data on the POP contamination status of wild shellfish, a dearth of Human Health Risk Assessments on local marine species, a lack of POP testing outside of harbours, and the absence of broad-scale testing for a wide range of POP contaminants.

The aims of this research were therefore to assess and quantify the POP contamination status of OCPs, OPPs, PAHs, and PCBs in wild mussels (*Mytilus galloprovincialis*, *Choromytilus meridionalis*, and *Perna perna*) from the coastline of the Western Cape Province of South Africa, and to use the data from the baseline study to perform a Human Health Risk Assessment on the potential non-carcinogenic and carcinogenic impacts of POPs in wild mussels for human consumption.

## 2. Materials and Methods

**Figure 1:**
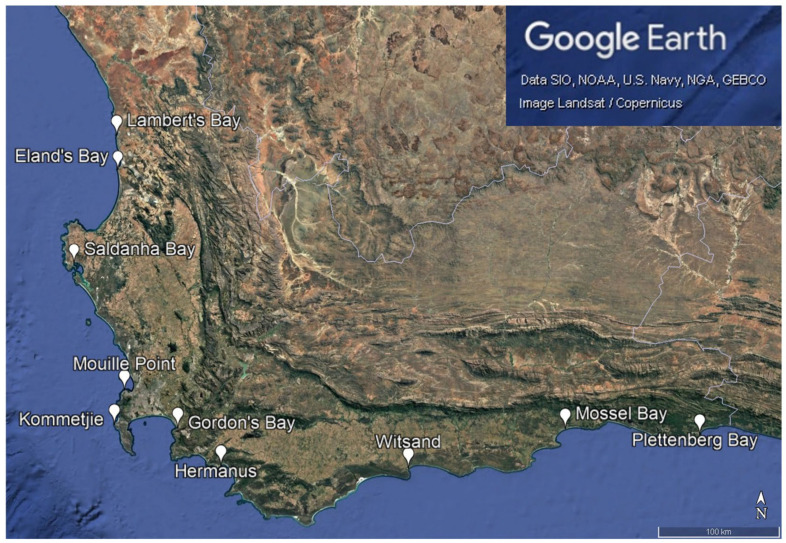
The 10 sampling sites in the Western Cape Province of South Africa from which wild mussels were collected in March and April 2019 (see Table 1 for location co-ordinates).

Sample collection: Wild, adult *Mytilus galloprovincialis*, *Perna perna,* and *Choromytilus meridionalis* mussels of 60–91 mm in length (comparable to harvest size in local aquaculture facilities) were collected at low tide over a 2-week period in March and April 2019 from 10 sites located along the Western Cape Province coastline (detailed in Figure 1). Mussels were collected by hand and stored in plastic polyethylene bags in a sealed cooler box with ice until they could be frozen (maximum 6 h from collection time). Once frozen, mussels were transported to the −20 °C freezer in the Meat Laboratory of the Animal Sciences Building, Stellenbosch University, where they were stored until analysis.

Sample preparation: Frozen mussels were thawed for 24 h prior to preparation. Whole mussels were weighed individually; subsequently, the whole meat (including all soft tissues, gonads, adductor muscles, and the digestive tract) was separated from the shell, and both parts were weighed and recorded. Mussels were not separated into body compartments (gonadal, feeding, digestive, etc.) as they are typically eaten whole; therefore, the whole mussel meat must be evaluated to correctly assess risk to consumers. Multiple individual mussels were then grouped into ‘pooled samples’ (or repeats). This method is common for pollutant research on smaller species, as an individual mussel is rarely large or heavy enough for analytical requirements after drying, and this practice helps reduce the high inter-individual variability [44,45,46]. Three repeats were created per location (from 12 to 15 mussels per location, meaning 4–5 mussels per repeat), and these repeats were then re-frozen at −20 °C. The frozen mussels were freeze-dried for approximately ≈72 h, before being blended to a homogenous powder [45,47]. Samples were weighed before and after the freeze-drying procedure to calculate exact conversion factors for each sample, rather than using a mean or estimated dry matter value.

Gas Chromatography Tandem Mass Spectrometry (GC-MS/MS) Analysis: The analytical methodology used for the GC-MS/MS analysis of POPs within mussels followed that of [42]. Restek Calibration Standards were used to establish six-point GC calibration curves for limits of quantification and detection of all analytes, at concentrations of 0.5, 1, 2.5, 5, 10, and 50 ppb. Each analytical run (n = 27 samples) included 5 randomly selected repeated samples for the purposes of quality control and assurance. The Restek Calibration Standard for 16 PAHs included naphthalene, acenaphthylene, acenaphthene, fluorene, phenanthrene, anthracene, fluoranthene, pyrene, chrysene, benz(a)anthracene, benzo(b,k)fluoranthracene, benzo(a)pyrene, indeno(1,2,3)pyrene, dibenzo(a,h)anthracene, and benzo(ghi)perylene [at 200 µg mL^−1^, in methanol: benzene: methylene chloride (80:1.25:178.75)]. The PCB standard included 11 PCB congeners, namely 18, 28, 44, 52, 70, 110, 118, 138, 149, 153, and 180 (at 500 µg mL^−1^ in acetone). The OCP standard included 23 pesticides, including chloroneb; hexachlorobenzene; alpha-, beta-, gamma-, and y-HCH (hexachlorocyclohexane); heptachlor; acetochlor; aldrin; heptachlor epoxide; alpha-chlordane; tans-nonachlor; endosulfan I and II; endosulfan sulphate; 4,4-DDT, -DDE, and -DDE; dieldrin; chlorobenzilate; endrin; and cis- and trans-permethrin (at 500 µg mL^−1^ in acetone). The limits of quantification and detection for all analytes were determined to be 0.5 ppb.

The Quick, Easy, Cheap, Efficient, Rugged, and Safe (QuEChERS) method of pollutant analysis was used within this research. Approximately 1 g of freeze-dried, homogenised sample was weighed out into a labelled, empty 50 mL QuEChERS centrifuge tube and the weight was recorded (0.000 g). A total of 9 ml Milli-Q water was added, and the sample was vortexed (please note that the original QuEChERS methodology required 5 mL Milli-Q water, plus 4 mL Milli-Q water to account for water loss during the freeze-drying process). Next, 10 mL of acetonitrile (H3CN) was added to the QuEChERS centrifuge tube and the mixture was vortexed. The first packet of QuEChERS solution (NaCl 1.5 g and MgSO_4_ 6 g; CAT: MS-MG5051-1, Lot: M02730) was then added to the QuEChERS centrifuge tube. The solution became warm and formed clumps, and samples were subsequently sonicated in a water bath for 30 min. After sonication, samples were centrifuged for 3 min at 5000 rpm (or until clear separation of acetonitrile layer). A total of 5 ml of the acetonitrile layer were then transferred into labelled 15 mL QuEChERS tubes containing the QuEChERS ‘clean-up’ powder (300 mg PSA, 150 mg C18, and 900 mg MgSO_4_). The 15 mL QuEChERS centrifuge tubes were then vortexed for five minutes, before being centrifuged for 3 min at 5000 rpm. Next, 1 mL of the top layer was transferred into an Eppendorf tube, with 20 µL semi-volatile internal standard mix (100 ppb) being added; then, the samples were vortexed. All liquid from the Eppendorf tube was then transferred to a 2 mL glass GC vial. These samples were then dried overnight using nitrogen and were reconstituted with 1 mL toluene before analysis. One µL of each sample was injected into a Thermo TSQ 8000 triple quadrupole MS (operated in selected reaction monitoring mode) (Thermo Scientific, Waltham, MA, USA; www.thermofisher.com), with the separation of the POPs being performed via Rxi 1310 gas chromatography coupled with a non-polar Rxi-5Sil MS with an integraGuard capillary column (15 m, 0.25 mm ID, 0.25 µm film thickness). The source temperature of ionisation was set to 250 °C and an emission current of 50 µA was utilised with argon collision.

Statistical Analyses: All results are presented in ng g^−1^ dry weight (d.w.), except for the values within the risk assessment, which were converted to wet weight (w.w.) to allow for a direct comparison to the legal limits for POPs in foodstuffs set by local and international regulatory bodies. It is important to note that many environmental and human risk assessment studies report data as dry weights, with the final wet weight concentrations of TEs typically being approximately five times lower than the dry weight [48,49].

The experimental design was completely randomised, with the main effects being location and species. All data were analysed with Microsoft Excel’s XLSTAT [Addinsoft (2023) statistical and data analysis solution, New York, USA [https://www.xlstat.com/ (accessed on 7 March 2023)]. A complication associated with the collection of wild mussels is that they often do not overlap in location; this resulted in there being only one species available per location within the current research, thereby complicating statistical analysis and making it difficult to analyse interaction terms between main effects. Species was therefore analysed first and determined to have no significant effect on the results by performing an ANOVA for location, with species as a grouping variable. A one-way ANOVA was then performed on the data to determine the potential effects of location on POP contamination. The data were checked for normality using the Shapiro–Wilk test, and, if needed, data were log-transformed to achieve the normality of the residuals. Levene’s test was used to check for the homoscedasticity of the data, and when data were determined to be heteroscedastic, Welch’s statistic was used in place of the F-statistic. Post hoc Bonferroni pairwise comparison tests were used to identify significant differences in POP contamination between sampling locations. All statistical analyses were performed on the dry weight concentrations of POPs (in ppb).

Human Health Risk Assessment: Human Health Risk Assessments (HHRAs) are typically divided into two sections—non-carcinogenic and carcinogenic health impacts. When contaminants are assessed for their toxicity by the USEPA, European Food Safety Authority (EFSA), and WHO, the potential carcinogenic and non-carcinogenic impacts are assessed using different limits and equations [50,51,52]. Contaminants that have been assessed for their non-carcinogenic health risks are often not subjected to corresponding carcinogenic health risk assessments if there is not enough available research to make accurate recommendations [50].

Non-carcinogenic HHRA:

The Estimated Daily Intakes (*EDIs*) of each PAH, PCB, and organochlorine pesticide (OCP) were calculated for adults and children consuming 250, 500, or 1 000 g of mussel meat per month (equivalent to one, two, or four portions of mussels a month, respectively), using the formula from [32,53], as follows:

$$EDI=\frac{CR\times Ci}{BW}$$

where *CR* is the consumption rate (in mg d^−1^), *Ci* is the POP contaminant concentration (in mg kg^−1^ w.w.), and *BW* is the human bodyweight (simplified to 70 kg for adults and 15 kg for children). *EDIs* were calculated for both the mean individual POP contamination detected for the whole coastline (n = 27) and the maximum single contamination value detected in any sample (n = 1) in order to assess all potential risk levels. These EDIs (in milligrams per kilogram bodyweight per day, or mg kg^−1^ bw d^−1^) were then compared to the *RfD* (in mg kg^−1^ bw d^−1^) using the following equation for the Hazard Quotient (*HQ*) and Total Hazard Index (*THI*) [32,53,54]:

$$THI=\Sigma HQ=\frac{EDI}{RfD}$$

The *THI* is a summary of all the individually calculated *HI* values [32]. If the value of the *THI* or *HQ* is > 1, then there are potential non-carcinogenic human health risks from chronic exposure to that PAH or combination of PAHs through consumption of the foodstuff (e.g., mussels). The available RfDs per group of POPs are as follows.

PAHs: Six PAHs had established RfDs, namely acenaphthene (0.06 mg kg^−1^ bw d^−1^), anthracene (0.3 mg kg^−1^ bw d^−1^), fluorene and fluoranthene (0.04 mg kg^−1^ bw d^−1^), naphthalene (0.02 mg kg^−1^ bw d^−1^), and pyrene (0.03 mg kg^−1^ bw d^−1^) [50]. There is also an RfD available for benzo(a)pyrene (BaP) (0.003 mg kg^−1^ bw d^−1^), but that is not included within this research as BaP was not detected in any of the mussel samples.

PCBs: Five detected PCBs were assessed using the RfDs for Aroclor 1016 and Aroclor 1254. Aroclors are commercial mixtures of PCBs that were used in human industry before being banned and will typically have different compositions to environmental mixtures of PCBs [50]. The increasing degrees of chlorination of a PCB mixture are related to the increasing toxicity of the mixture; Aroclor 1016 is a PCB mixture with no chlorinated dibenzofurans [also known as non-dioxin-like (NDL) PCBs], and is therefore considered less toxic than Aroclor 1254, which contains polychlorinated dibenzofurans [50,55]. The established oral RfDs are 7 E^−5^ mg kg^−1^ bw d^−1^ for Aroclor 1016 and 2 E^−5^ mg kg^−1^ bw d^−1^ for Aroclor 1254 [50]. Due to there being no dioxin-like PCBs detected within mussels from the current research (no PCDDs or PCDFs), the RfD for Aroclor 1016 was used for this risk assessment.

OCPs: Of the two OCPs [β-hexachlorocyclohexane (HCH) and chlordane] detected in wild mussels, only chlordane had an available oral RfD (5 E^−4^ mg kg^−1^ bw d^−1^) from the USEPA [50]. Beta-HCH has not been assessed for oral toxicity, but γ-HCH has an established RfD of 3 E^−4^ mg kg^−1^ bw d^−1^, which was used to assess the risk within the parameters of this research [50]. The accuracy of this risk assessment is assumed to be low but provides a method of comparison and could potentially help identify locations with excessive HCH concentrations in the mussels.

Carcinogenic HHRA

Carcinogenic HHRAs were performed for POP contaminants with cancer-causing effects in humans for which there were available Cancer Slope Factors (CSFs). Cancer Slope Factors were not available for any of the PAHs detected within the mussels in this study [50]. Polychlorinated biphenyls, as a class, have been assessed and their Cancer Slope Factor has been established as 2 mg kg^−1^ bw day^−1^ (upper-bound slope factor) for food chain exposure [50]. For OCPs, chlordane and β-Hexachlorocyclohexane have been assessed for carcinogenicity, with the CSFs established as 0.35 and 1.8 mg kg^−1^ bw day^−1^, respectively [50]. The carcinogenic risk is established using the following formula:

CR=EDI×CSF

where *CR* is the carcinogenic risk level and *CSF* is the Cancer Slope Factor. The threshold for cancer risk was set to the public screening criteria of one in one million; a carcinogenic risk value less than 10^−6^ is an acceptable level of risk. Values between 10^−6^ and 10^−4^ fall into an area of concern, while values greater than 10^−4^ represent an unacceptable level of risk [53,56].

## 3. Results

### 3.1. Wild Sample Collection

Of the analysed mussels, 49 were *Mytilus galloprovincialis*, 41 were *Choromytilus meridionalis*, and 21 were *Perna perna*. The local *C*. *meridionalis* dominated in Lambert’s Bay, Mouille Point, and Gordon’s Bay, while the invasive *M*. *galloprovincialis* was most abundant in Eland’s Bay, Saldanha Bay, Kommetjie, and Hermanus. *P*. *perna* were only present in Witsand and Mossel Bay (Table 1). Due to differences in habitat preference, not all species were present at all sampling locations, and the results of the statistical analyses are therefore interpreted with caution.

The maximum, minimum, and mean (± standard error) concentrations of all detected POPs in wild Western Cape Province mussels are detailed in Table 2. Eleven different PAHs were detected—acenaphthene, acenaphthylene, anthracene, benzo(a)anthracene, benzo(ghi)perylene, chrysene, fluoranthene, fluorene, phenanthrene, pyrene, and naphthalene. A total of 5 PCB congeners were detected in wild South African mussels; these included PCBs 8, 18, 28, 44, and 52 (Table 2). Only two OCPs were detected in the wild coastal mussels, namely chlordane and β-HCH.

### 3.2. Polyaromatic Hydrocarbons by Location (PAHs)

**Figure 2:**
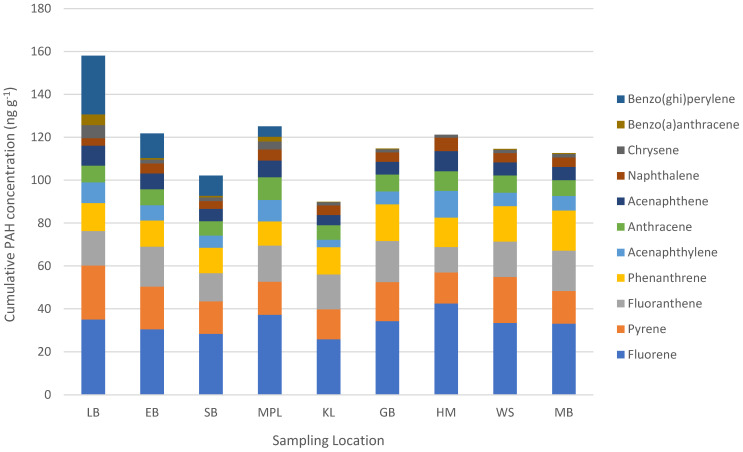
Mean PAH concentrations (ng g^−1^ d.w.) in wild mussels from nine locations along the coastline of the Western Cape Province, South Africa (2019).

As depicted in Figure 2, the PAH with the highest concentration in wild mussels from all locations was fluorene (min: 25.8 ng g^−1^ d.w. in Kommetjie; max: 42.5 ng g^−1^ d.w. in Hermanus), followed by lower concentrations of pyrene (min: 14.0 ng g^−1^ d.w. in Kommetjie; max: 25.1 ng g^−1^ d.w. in Lambert’s Bay), fluoranthene (min: 11.9 ng g^−1^ d.w. in Hermanus; max: 19.2 ng g^−1^ d.w. in Gordon’s Bay), and phenanthrene (min: 11.2 ng g^−1^ d.w. in Mouille Point; max: 18.7 ng g^−1^ d.w. in Mossel Bay). There were typically lower concentrations of acenaphthylene (min: 3.5 ng g^−1^ d.w. in Kommetjie; max: 12.5 ng g^−1^ d.w. in Hermanus), anthracene (min: 6.6 ng g^−1^ d.w. in Saldanha Bay; max: 10.6 ng g^−1^ d.w. in Mouille Point), acenaphthene (min: 4.8 ng g^−1^ d.w. in Kommetjie; max: 9.4 ng g^−1^ d.w. in Hermanus), and naphthalene (min: 3.5 ng g^−1^ d.w. in Lambert’s Bay; max: 6.3 ng g^−1^ d.w. in Hermanus) (Figure 2). The three POPs with the lowest concentrations in wild mussels were chrysene (min: 1.4 ng g^−1^ d.w. in Hermanus; max: 6.1 ng g^−1^ d.w. in Lambert’s Bay), benz(a)anthracene (NF in Hermanus samples; min: 0.17 ng g^−1^ d.w. in Kommetjie; max: 5.0 ng g^−1^ d.w. in Lambert’s Bay), and lastly benzo(ghi)perylene, which was only detected in mussel samples from four of the analysed locations (min: 4.9 ng g^−1^ d.w. in Mouille Point; max: 27.3 ng g^−1^ d.w. in Lambert’s Bay), meaning there were significant statistical differences in concentration between locations (*p* ≤ 0.05).

While data were also analysed per location for PCBs 8, 18, 28, 44, and 52, as well as the OCPs β-HCH and chlordane, there were no significant differences (*p* ≥ 0.05) in contaminant concentrations between locations, and these graphs have therefore not been included in this manuscript. For those who are interested, these graphs are available in the Supplementary Materials (Figures S1–S3).

### 3.3. Risk Assessment of POPs

Please note that data in this section are presented in wet weight (w.w.) for the ease of comparison to legal limits and for risk assessment calculations; no statistical analyses were performed on the data within the risk assessment section.

Non-carcinogenic risk assessment of PAHs: A direct comparison of PAHs with limits set by the European Commission was performed first. For PAHs in foodstuffs, a limit of 5 ng g^−1^ has been set for benzo(a)pyrene alone, and a limit of 30 ng g^−1^ was set for the sum of benzo(a)pyrene, benz(a)anthracene, benzo(b)fluoranthene, and chrysene [57]. No benzo(a)pyrene was detected in any of the mussel samples and of the other contaminants mentioned above; only benzo(a)anthracene and chrysene were detected. When combined, the mean concentration of benzo(a)anthracene and chrysene (0.2 ± 0.06 and 0.4 ± 0.06 ng g^−1^ w.w., respectively) was ~0.6 ng g^−1^ w.w., which is far below the 30 ng g^−1^ w.w. limit set by the EC [57]. Even when combining the maximum detected benzo(a)anthracene and chrysene concentrations (1.6 and 1.7 ng g^−1^ w.w., respectively), the total amount of contamination (~3.2 ng g^−1^ w.w.) is almost ten times smaller than the limit. This initial result implies that PAH contamination levels in wild mussels from the Western Cape Province coastline are low and unlikely to pose health risks to human consumers.

The results of the EDI, HI, and THI calculations for adults and children consuming 250, 500, or 1000 g mussel meat per month (g m^−1^) are presented in Table 3 below. Altogether, 6 of the 11 PAHs that were detected within mussels had RfDs available on the IRIS USEPA, namely acenaphthene, anthracene, fluoranthene, fluorene, naphthalene, and pyrene. In order to present a range of human health risks, the risk assessment process was performed on both the mean PAH concentrations detected for the entire Western Cape Province coastline and the highest detected concentration in wild mussels along the coast (Table 3). The EDIs calculated for acenaphthene, anthracene, fluoranthene, fluorene, naphthalene, and pyrene were consistently below the RfDs set for these contaminants in food for both adults and children for all rates of consumption (Table 3). The Hazard Quotients (HQs) were all below the threshold for risk (<1) for all PAH concentrations and consumption levels, indicating that the individual PAHs do not pose health risks to human consumers of wild mussels. For adults, the THI (sum of HQs) was 0.048–0.21 for average PAH concentrations and 0.10–0.41 for maximum PAH concentrations, which is below the threshold for human health risk (<1). For children, the THI values were 0.23–0.97 for mean PAH concentrations and 0.45–1.93 for maximum PAH concentrations in wild mussels from the Western Cape Province coastline (Table 3). The threshold for risk was approached for children consuming wild mussels with mean PAH concentrations at a rate of 1000 g m^−1^, as well as for children consuming wild mussels with maximum PAH concentrations at a rate of 500 g m^−1^. The threshold for risk was exceeded by children consuming 1000 g m^−1^ of mussel meat from areas with maximum PAH concentrations.

Carcinogenic risk assessment of PAHs: There was no carcinogenic risk assessment performed for PAHs, as the PAHs detected in this research did not have available Cancer Slope Factors.

Non-carcinogenic risk assessment of PCBs: A direct comparison of PCB concentrations in wild mussels to the standards set by the European Commission was performed first. The EC has set a limit of 6.5 pg g^−1^ (0.0065 ng g^−1^) for dioxins and dioxin-like PCBs, but no dioxin-like PCBs were detected during the current research. The EC also set a limit for fishery products of 75 ng g^−1^ for the six indicator NDL-PCBs (congeners 28, 52, 101, 138, 153, and 180), which typically represent >50% of the PCB congeners found in foodstuffs [57]. The five PCBs detected in wild mussels were PCB 8, 18, 28, 44, and 52, meaning that the only two indicator PCBs detected in wild mussels were congeners 28 and 52. The combined mean contamination of wild mussels by PCB 28 (mean: 1.2 ± 0.11 ng g^−1^ w.w.) and PCB 52 (mean: 0.8 ± 0.03 ng g^−1^ w.w.) was ≈2.0 ng g^−1^ w.w., which is far lower than the 75 ng g^−1^ limit set for the six indicator PCBs. Even when taking the highest single recorded concentrations of PCBs 28 and 52 (3.8 and 1.1 ng g^−1^ w.w., respectively), in combination they still only reach 4.9 ng g^−1^ w.w., which is still far lower than the limit set by the European Commission. These results indicate that there is likely very little non-carcinogenic risk posed to wild mussel consumers from NDL-PCBs.

The results of the EDI, HQ, and THI calculations for mean and maximum PCB concentrations in wild mussels from the Western Cape Province coastline are presented in Table 4. The EDIs for all tested PCBs (namely congeners 8, 18, 28, 44, and 52) and rates of consumption [250, 500, or 1000 g per month (g m^−1^)] were greater than the RfD for Aroclor 1016, which is 7 E^−5^ mg kg^−1^ bw day^−1^ (Table 4). For the HQs and THIs, all calculated values for adults and children were >1 (the threshold for risk) for all consumption rates [53,58]. It must be noted that the RfD for Aroclor 1016 was established for commercial mixtures of PCBs used in industrial settings (prior to being banned) and is used here to allow for a comparison and to assist in a more comprehensive risk assessment process, despite the author being aware of Aroclor mixtures generally differing from environmental combinations of PCBs [50].

#### Carcinogenic Risk Assessment

The results of the cancer risk assessments are presented in Table 5, Table 6 and Table 7 below. Values within the area of concern (between 10^−4^ and 10^−6^) remain in black text, and values that are considered to have an unacceptable cancer risk (>10^−4^) are highlighted in red. All of the tested PCBs were identified to have carcinogenic risk levels that exceeded the “unacceptable” risk level for both adults and children at any level of wild mussel consumption (Table 5).

Non-carcinogenic and carcinogenic risk assessments of OCPs

Chlordane has an oral RfD of 5 E^−4^ mg kg^−1^ bw day^−1^ and a CSF of 0.35 mg kg^−1^ bw day^−1^ [50]. The results of the non-carcinogenic and carcinogenic risk assessments of chlordane in wild mussels are presented in Table 6, with values that exceed safe levels highlighted in red. Hazard Quotients and THIs for non-carcinogenic risk were >>1 for both adults and children consuming 250–1000 g of wild mussel meat per month (g m^−1^) from the Western Cape Province coastline (Table 6). The cancer risk values calculated for mean and maximum chlordane concentrations in wild mussels were above the unacceptable level of risk (10^−4^) for all levels of consumption by adults and children. Even the lowest level of wild mussel consumption (250 g m^−1^) resulted in unacceptable levels of non-carcinogenic and carcinogenic human health risks.

There is no RfD available for β-HCH, but an RfD of 0.0003 mg kg^−1^ bw day^−1^ has been established for γ-HCH, and a cancer risk assessment has been performed for β-HCH, which determined a CSF of 1.8 mg kg^−1^ bw day^−1^ [50]. The results of the non-carcinogenic and carcinogenic risk assessments of β-HCH are presented in Table 7. For adults, mean β-HCH concentrations within wild mussels resulted in EDIs that were lower than the RfD for consumption rates of 250–500 g m^−1^, indicating that this would not have non-carcinogenic health risks for adult humans. For maximum β-HCH concentrations, only adults consuming ≥ 250 g m^−1^ would be avoiding non-carcinogenic health risks. For all other adult consumption levels, as well as for all child consumption rates, the HQ values exceeded the threshold for risk (>1) and the cancer risk was in the unacceptable range. The results presented in Table 7 suggest that there is no safe level of wild mussel consumption with regard to β-HCH for children (due to non-carcinogenic and carcinogenic risks), and adults should consume < 500 g m^−1^ of wild mussels to avoid non-carcinogenic health risks and <250 g m^−1^ to avoid potential carcinogenic health risks.

## 4. Discussion

Polyaromatic hydrocarbon concentrations were consistent along the coastline for fluorene, pyrene, fluoranthene, phenanthrene, acenaphthylene, acenaphthene, anthracene, naphthalene, and chrysene (Figure 2). Prior local research into the temporal fluctuations of three PAHs (fluorene, fluoranthene, and benz(a)anthracene) found these contaminants to have consistent concentrations in mussels over a two-year study period [42]. PAHs persist in the fat of the animals that ingest them [8], and the relatively stable PAH concentrations may suggest that mussels are experiencing similar levels of exposure along the Western Cape Province coastline (ubiquitous contamination). Mussels have a limited biotransformation capacity for PAHs [59,60], and it is therefore unlikely that the mussels themselves are regulating PAH concentrations within their meat. The two PAHs with varying concentrations along the coastline were benzo(ghi)perylene and benz(a)anthracene (Figure 2). Benz(a)anthracene was only detected in Lambert’s Bay, Eland’s Bay, Saldanha Bay, and Mouille Point mussel samples, i.e., only sampling locations on the West Coast of the Western Cape Province. Benzo(ghi)perylene was detected in mussels from all locations except Hermanus. The notable fluctuations of these PAH contaminants between locations suggests that there may be local inputs of these contaminants into certain areas of the coastline. It is difficult to determine whether this fluctuation in contaminants is entirely species- or location-related, because both wild *C*. *meridionalis* and *M*. *galloprovincialis* mussels dominated the West Coast in different locations (Table 1). Benzo(ghi)perylene and benz(a)anthracene are released into the environment via the incomplete combustion of mostly organic materials such as wood, coal, oil, and plastic [61,62,63]. This suggests that the sampling locations with elevated concentrations of these contaminants may have higher exposure levels due to improper waste management (burning of waste) or heavy industry.

Prior research on POPs in farmed mussels from Saldanha Bay found only five PAHs [42] whereas the current research found eleven different PAHs in mussels from most locations. In the research from 2019, the average fluorene and fluoranthene concentrations were less than half that of the current research (Table 2), though the maximum detected concentrations were similar. The average pyrene contamination was higher in the current study, though the maximum detected pyrene concentrations were far higher in the former study [42]. Benz(a)anthracene means were alike for both studies, though the maximum concentrations were three times greater in the current research. These results suggest that wild mussels may be exposed to a wider range and higher concentration of PAH contaminants than farmed mussels. Differences in PAH contamination between caged, transplanted mussels and wild mussels have been detected before [64]. A potential reason for differences in contamination is that farmed mussels are typically situated in locations with high water circulation and food availability, leading to faster growth rates and less exposure to contaminants over a shorter lifetime. Wild mussels generally grow far slower due to their preferred intertidal habitats causing them to be tidally exposed to air for up to 12 h a day, resulting in lower rates of feeding [65] and leading to a longer lifespan over which lipophilic contaminants such as POPs can accumulate. While increased rates of feeding could potentially mean that farmed mussels are more consistently exposed to contaminants in the water column and therefore might have had higher PAH concentrations than wild samples, this does not appear to be the case.

At least four PAHs [pyrene, benz(a)anthracene, benzo(ghi)perylene, and chrysene] had accumulation patterns that showed high POP contamination in mussels from Lambert’s Bay, which is the northernmost location on the West Coast (Figure 1). The clear pattern cannot be extrapolated further along the coast, and it cannot be assumed that PAH pollution increases exponentially up the coast, but it is a contamination concern. The PAH contamination may be highest in Lambert’s Bay mussels and then decreases again along the coastline of the Northern Cape Province, but this cannot be known until mussels from the Northern Cape are investigated for these pollutants. Elevated concentrations of benz(a)anthracene and benzo(ghi)perylene in West Coast samples (when compared to samples from the South Coast) resulted in the cumulative concentrations of POPs being higher on the West Coast than in other sampling locations.

To discern the potential source of coastal PAH contamination, the total concentrations of low-molecular-weight (LMW) PAHs (<4 aromatic rings) were compared with the total concentration of high-molecular-weight (HMW) PAHs (≥4 aromatic rings) [5,66]. The LMW PAHs within the current research were naphthalene, acenaphthylene, acenaphthene, fluorene, phenanthrene, and anthracene, while the HMW PAHs were fluoranthene, pyrene, benz(a)anthracene, benzo(ghi)perylene, and chrysene. The PAH with the highest concentration in wild mussels was fluorene (Figure 2), and the total LMW PAH concentrations were 1.7 times higher than the total HMW PAH concentrations. When looking at LMW/HMW PAH ratios for individual locations, LMW PAHs were dominant in Mouille Point, Kommetjie, Gordon’s Bay, Hermanus, and Mossel Bay, and HMW PAHS were greater in Lambert’s Bay, Eland’s Bay, Saldanha Bay, and Witsand. Locations with greater concentrations of LMW PAHs indicate that pollution is largely coming from petrogenic sources (i.e., fossil fuels), while those with more HMW PAHS likely come from more pyrolytic sources (i.e., incineration processes) [5,67,68].

Human Health Risk Assessment of PAHs: Direct comparisons of wild mussel PAH concentrations [benzo(a)pyrene, benz(a)anthracene, benzo(b)fluoranthene, and chrysene] to the limits set for bivalve molluscs by the European Commission showed that concentrations were very low and likely pose little risk of harm to consumers. A non-carcinogenic HHRA was performed for 6 of the 11 PAHs detected within wild mussels from the Western Cape Province (Table 3). The results of this HHRA corroborated the results of the direct comparison. The EDI, HI, and THI values showed that most levels of mussel consumption [250–1000 g per month (g m^−1^)] posed little risk to adult or child consumers, even when tested cumulatively (Table 3). The risk to children was established for the cumulative total of maximum PAH concentrations (THI) in wild mussels at the highest consumption rate of 1000 g m^−1^. It is therefore advisable that children avoid consuming > 500 g m^−1^ wild mussels harvested from areas where there may be increased PAH pollution (e.g., harbours or industrial areas). This indicates that while PAHs are consistently present in wild mussels from the Western Cape Province coastline, these contaminants generally have low enough doses that they are unlikely to pose health risks to regular consumers of wild mussels.

Polychlorinated biphenyls are a range of chemicals (209 congeners) used in the electrical industry as heat exchange fluids but have been banned in many countries since the 1970s [1]. The potential carcinogenic and immune system impacts of PCBs in foodstuffs led them to be included in the “Dirty Dozen” list of the Stockholm Convention on POPs [1,14]. PCBs have never been produced in South Africa but are still used within the petrochemical, mining, transport, and energy industries [13]. As a signatory of the Stockholm Convention since 2004, South Africa has classified imports of PCBs as “Prohibited and Restricted” and is in the process of phasing out all PCB oils and oil-containing equipment [13].

In the current research, the five identified PCBs (congeners 8, 18, 28, 44, and 52) had no statistically significant differences in concentration around the Western Cape Province coastline, indicating that neither location nor species had an impact on PCB concentrations in wild mussels. The PCB with the highest concentration in wild mussels was PCB congener 18, followed by PCB 8, PCB 28, PCB 44, and finally PCB 52, which had the lowest overall concentrations. Prior research into PCB contamination in mussels transplanted into four South African harbours found PCB contamination to be present in all locations [41]. When compared to local research on the PCB contamination of mussels, the total PCB content in the current research was greater than the concentration of PCBs in farmed mussels from Saldanha Bay [42] and lower than within mussels from Port Elizabeth (Gqeberha) [39,40]. In South Africa, PCB concentrations appear to be lowest in farmed mussels, increase in concentration for coastal mussels, with peak concentrations being found in harbour mussels (where there is high contaminant exposure and low water circulation). Similarly to the pattern seen for PAHs, this makes biological sense because local research has shown *C*. *meridionalis*, *M*. *galloprovincialis*, and *P*. *perna* mussels grow faster in areas with higher water circulation, with growth being progressively slowed by increasing levels of tidal exposure [65]. As discussed for PAHs, wild mussels likely have far longer lifespans than their farmed counterparts, allowing for more time for pollutant bioaccumulation.

The total PCB concentrations in the current research (34.4 ng g^−1^ d.w. or 6.3 ng g^−1^ w.w.) were similar to those from local mussel research in harbours and restricted water bodies [39,40], as well as caged and native mussels from Norway [64], Croatia [69], the Adriatic Sea [7], and Turkey [70], in addition to shellfish from China [53]. The total PCB concentrations within the current research were typically lower than those detected for the coast of South Korea [71], the Danish Straits and Baltic Sea [72], the United States of America [73,74], and the Chilean coastline [75].

Much of the research on the accumulation of PCBs in mussels has found them to have elevated concentrations of higher chlorinated biphenyls (congeners 138, 153, and 180) [7,39,40,41,70]. The current research does not follow this trend, with exclusively lower chlorinated biphenyls (congeners 8, 18, 28, 44, and 52) being detected. This suggests that the composition of PCB contamination of wild, coastal mussels differs significantly from that of mussels from farming locations and harbours.

Human Health Risk Assessment of PCBs: The direct comparison of the concentrations of six indicator NDL-PCBs (28, 52, 101, 138, 153, and 180) to the 75 ng g^−1^ w.w. limit set for these contaminants in fishery products [57] found individual and cumulative PCB concentrations within local wild mussels to be very low and of little risk to consumers (2.03 ng g^−1^ w.w.). This remained true even when taking the single highest detected PCB concentrations into account (4.88 ng g^−1^ w.w.).

However, the results of the non-carcinogenic HHRA (Table 4) showed that EDIs were consistently above the RfD for NDL-PCBs (Aroclor 1016), with both HI and THI values being significantly greater than the threshold for risk (>1). The carcinogenic HHRA had similar results, with all levels of consumption in both adults and children resulting in unacceptable cancer risks (>10^−4^), and risks increased proportionally with increasing consumption rate or decreasing child bodyweight (Table 5). It is noteworthy that the USEPA has conceded that using the RfD for Aroclors as an RfD for environmental PCBs may have variable accuracy, because environmental compositions of PCBs (which are somewhat broken down by natural processes) can differ from those in commercial Aroclor mixes. Due to the lack of an alternative for comparison, the RfD for Aroclor 1016 was used within the current research because it does not contain any dioxin-like PCBs, and no dioxin-like PCBs were detected in the wild mussels from the Western Cape Province. If dioxin-like PCBs had been detected in the mussels, the RfD for Aroclor 1254 would have been used instead, as this RfD considers the increased human health risk posed by dioxins within foodstuffs. It can therefore only be stated with a medium degree of confidence that PCB concentrations in mussels currently pose potential non-carcinogenic human health risks to wild mussel consumers. It can, however, be stated with high confidence that PCBs in wild mussels from the Western Cape Province coastline likely pose carcinogenic health risks to regular consumers.

Two organochlorine pesticides were detected in wild mussels—β-HCH and chlordane. Hexachlorocyclohexanes (HCHs) are a class of insecticides formerly used on fruit and vegetable crops, in forestry, in seed treatment, and to control lice and scabies [50,76]. Technical HCH is a mix of isomers that includes α-HCH (~60–70%), β-HCH (~5–12%), γ-HCH (~10–15%), δ-HCH (~6–10%), and ε-HCH (~3–4%) [77]. β-HCH has been theorised to be the most lipophilic and therefore most persistent in nature [78]; only β-HCH was detected within mussels in the current research, suggesting that the concentrations detected are from historical pollution (before South Africa signed the Stockholm International Convention on POPs) and are unlikely to be from present sources of HCH. Concentrations of β-HCH were consistently low in wild mussels from the coastline of the Western Cape Province, and significant differences in contamination were not evident between sampling locations. This environmental contaminant is considered to be both widespread and poorly researched [78]. Since it is known to be extremely lipophilic and present in mussels (the base of the marine food chain), it is advised that higher-trophic-level species should be assessed for the accumulation of β-HCH. The only other local research which has recently analysed and quantified HCH concentrations in the South African marine environment determined the sediments of Richard’s Bay Harbour (and surrounding areas) to exceed sediment quality guidelines and likely pose health risks to both the environment and consumers [31]. This study, like ours, emphasised the urgent need for further research into the OCP contamination of South African marine environments, which is a critically under-researched field in the country [18].

The POP with the highest concentration within the current study (by many orders of magnitude) was chlordane (Table 2). Chlordane is a broad-spectrum, agricultural pesticide formerly used to control termite infestations, but it was banned from use in the United States of America in 1988 after it was discovered to have both acute (neurological symptoms and gastrointestinal distress) and chronic (nervous system effects and cancer) health impacts on humans [50,79]. Chlordane was one of the first OCPs to be included in the “Dirty Dozen” list from the Stockholm Convention on POPs [1]. In South Africa, it has been listed as a “Group 1” hazardous substance that has required a special licence for local use since 2005 and is prohibited for use in agriculture [13].

Previous research quantifying OCPs in local mussels found chlordane to be present in farmed mussels from Saldanha Bay (mean: 1.2–2.3 ng g^−1^ d.w.; max: 6.7 ng g^−1^ d.w.) but the concentrations were far below those within the current research (Table 2) and were not considered to pose any health risk to farmed mussel consumers [42]. A potential reason for this difference is because Saldanha Bay has no large riverine inputs that could transport agricultural chemicals into the Bay ecosystem [80], and this is somewhat supported by the fact that wild mussels from Saldanha Bay in the current study had the lowest overall chlordane concentrations of any sampling location (Supplementary Material, Figure S3). The most likely explanation for the difference in contamination levels between the two studies is that the samples from the former study were farmed mussels, while the current samples are wild. As previously discussed for PAHs and PCBs, wild mussels grow far slower than their farmed counterparts from the same areas [65], resulting in wild mussels having more time for lipophilic contaminant exposure and accumulation. Farmed mussels have a more consistent exposure to food sources, resulting in greater bodyweights than in wild mussels; a heavier mussel may therefore have lower concentrations of contaminants in their meat due to increased mass having a dilution effect.

Human Health Risk Assessment of OCPs: The non-carcinogenic HHRA for β-HCH was performed using the RfD for y-HCH (due to the absence of a β-HCH-specific limit) and is therefore not considered to be health protective, but rather an indicator of potential contamination. The calculated Hazard Quotient (HQ) values were below the limit that denotes non-carcinogenic risk to consumers (<1) for adults ingesting 250–500 g m^−1^ of wild mussels with mean β-HCH concentrations and 250 g m^−1^ for wild mussels with maximum β-HCH concentrations (Table 7). For all other levels of adult consumption, as well as all child consumption rates, HQ and cancer risk values were greater than the thresholds for risk, indicating unacceptable levels of non-carcinogenic and carcinogenic risks. Keeping in mind that there is only medium confidence in the results of the non-carcinogenic HHRA, this tentatively indicates that adults can consume 500 g m^−1^ of wild mussel meat without serious non-carcinogenic health risks. At this rate of consumption, though, adults could experience negative health outcomes due to unacceptable cancer risks when consuming ≥ 250 g m^−1^ of mussel meat.

A direct comparison of the average (1945 ng g^−1^ w.w.) and maximum (3425 ng g^−1^ w.w.) chlordane concentrations within wild mussels with the protective limit recommended by the Food and Drug Administration of the United States of America (300 ng g^−1^ w.w.) showed that average and mean concentrations were 6 and 11 times greater than the recommended amount [81]. This result indicates that wild mussels are severely contaminated with chlordane, which is further confirmed by the non-carcinogenic and carcinogenic risk assessment results discussed below.

The non-carcinogenic and carcinogenic HHRAs showed HQ values and cancer risks (>10^−4^) to greatly exceed the safe limit (>1) for adults consuming ≥ 250 g m^−1^ mussel meat for both mean and maximum detected chlordane concentrations (Table 6). The calculated HQ and cancer risks increased drastically for children, showing the far greater risk experienced by those with lower bodyweights. The results in Table 6 indicate that there is no safe level of wild mussel consumption for adults or children in the Western Cape Province with regard to the persistent food chain contaminant known as chlordane.

With consistent oral exposure of 0.1–2 mg kg^−1^ bodyweight (bw) day^−1^, animal trials showed that chlordane caused kidney lesions, increased liver weight, and liver tumours, and at 4–10 mg kg^−1^ bw day^−1^, it delayed immunological development and even caused tremors [79]. Wild mussel consumers, particularly those harvesting mussels on a subsistence basis, may be orally exposed to these concentrations of chlordane on a regular basis (depending on sampling location); this food chain contaminant could therefore pose direct health risks to South African wild mussel consumers if not monitored.

However, these results must be interpreted with caution for a number of reasons. Firstly, while wild mussel harvesting is a popular hobby within South Africa, with thousands of recreational fishing licences sold per year, it is unlikely that consumers are ingesting contaminated mussels at a frequency and consistency that would put them at serious risk. A common saying in risk assessments is “the dose determines the poison”, but the frequency of exposure to the contaminant is also an important factor in negative health outcomes. Consumers ingesting wild mussels very infrequently or even switching between wild and farmed mussel options (which are closely monitored for POP contaminants), will be unlikely to experience the negative health outcomes linked with acute or chronic exposure to oral chlordane. Secondly, this is the first report of its kind to assess chlordane in wild mussels from South Africa, and the detected concentrations were extremely high when compared to other POP contaminants (Table 1). These high chlordane concentrations resulted in extremely high HQ and cancer risk values, which are difficult to translate into realistic consumer risk. The HQs used in risk assessments are index values that are calculated using EDIs and RfDs, both of which are estimated values. Reference doses (RfDs) for each contaminant are typically set using research performed on mice or other laboratory specimens (not humans); these values are set to be orders of magnitude lower than the concentration at which negative side effects will be experienced by general child and adult populations [50]. This means that while the extremely high risk values calculated within the current research do show potential risk to the regular wild mussel consumer from chlordane, this risk may be overestimated due to an overabundance of caution within the regulatory bodies which set oral contamination RfDs. The chlordane contamination values within the current research are only taken from the Western Cape coastline at one time for three mussel species; they could therefore represent an over- or under-estimation of risk from wild mussels to consumers. The researchers believe this further emphasises the urgent need for a Mussel Watch in South Africa, which would be able to monitor trends in POP contamination over time and confirm or refute the concerning results of the current research. Lastly, the POP concentrations within mussels from the current research were obtained from wet mussel weights (consistent with the literature), but most mussels are cooked before they are eaten, which is likely to change the concentration of POPs within them, as is the case with fish [82,83].

The carcinogenic and non-carcinogenic HHRAs for chlordane in wild mussels demonstrated that despite being banned for agricultural use within the country since 2005 [13], it is still present in marine ecosystems at levels that create both non-carcinogenic and carcinogenic risks for adults and children consuming mussels at even the lowest rates (250 g m^−1^). The results of this study raise major concerns and indicate that there may be no safe level of wild mussel consumption for coastal seafood harvesters due to this contamination (however, as discussed above, the current research may also represent an over-estimation of risk). There is a dire need for more investigation into this extremely toxic and persistent substance. Chlordane has a half-life of 30 years in the environment [84], meaning that the detected chlordane concentrations are likely from legacy inputs of this contaminant before it was banned for agricultural use [13]. The relative consistency of the chlordane contamination levels around the entire coastline of the Western Cape Province (Supplementary Material, Figure S3) also suggests that the detected concentrations may not be from novel introductions of this pesticide into the environment. South African rivers and estuaries have been identified as sinks of OCPs and other POP contaminants [30], and it is possible that even small rivers are still releasing chlordane-contaminated river water into the ocean, resulting in continuously high concentrations.

The major limitation of this research was cost. The analysis of POPs is complex and requires skilled technicians for operating GC-MS/MS equipment, which therefore drives up the analytical costs for researchers. Cost and equipment both limited the groups of POPs which could be analysed, as the laboratory could not obtain the necessary internal standards for PCDD or PCDF analysis within a reasonable budget. The limited number of samples that could be analysed per location (due to cost) may have negatively impacted the ability of statistical analysis procedures to find significant differences between location and species. This also limited the researcher’s ability to perform repeat sampling over the four seasons of a full year. Local research into farmed mussels from Saldanha Bay (performed over 2 years) found that most detected POPs did not fluctuate in concentration within mussels annually, with the exceptions of cis- and trans-permethrin and PCB congeners 18, 118, and 149 [42]. While this gives some evidence that many POP concentrations in mussels may not fluctuate significantly throughout a year, this research needs to be replicated on wild mussels to confirm whether seasonal fluctuations impact the safety of wild mussel consumption over a year. This further emphasises the need for a national Mussel Watch Programme in South Africa, which could repeatedly analyse mussels for priority contaminants like chlordane, and which could use the data obtained to give out advice to the public on the best harvesting times and least-polluted harvesting locations, or even close certain areas for wild mussel harvest when necessary. A major limitation to this research is also the general dearth of POP research, particularly in Africa [85,86]. The complexity of POP analysis, as well as the constant addition of novel environmental POPs via human industry, creates a situation where POPs are costly and difficult to analyse, and are typically analysed in small groups of contaminants (PAHs, PCBs, or OCPs) or avoided entirely.

## 5. Conclusions

The current study has confirmed that wild mussels from the Western Cape Province coastline are exposed to a wide variety of POPs that are retained in the meat of mussels. A total of eleven PAHs, five PCBs, and two OCPs were detected in wild mussels. Most POPs had consistent concentrations around the coastline, with the exception of benzo(ghi)perylene and benz(a)anthracene. The source of PAH contamination in mussels was determined to be both petrogenic and pyrolytic, indicating similar pollution contributions from fossil fuels and incineration, respectively. Direct comparisons found PAH and PCB concentrations in wild mussels to be within legal limits. The non-carcinogenic HHRAs performed for PAHs found no health risks associated with adults consuming approximately 250–1000 g m^−1^ of wild mussel meat and children consuming 250–500 g m^−1^. The non-carcinogenic and carcinogenic risk assessments of PCBs determined all consumption rates of wild mussels to result in health risks to adults and children. The HHRA on the OCP chlordane found very high levels of non-carcinogenic and carcinogenic human health risks to adults and children. For β-HCH, adults could consume 250–500 g m^−1^ mussel meat without non-carcinogenic health risks, though carcinogenic health risks were present for all consumption rates. Children had unacceptable non-carcinogenic and carcinogenic risks from all consumption rates. While these risks were established for specific locations and a single time period (2019), the extreme health concerns identified and the persistently elevated levels of legacy POPs such as chlordane indicate that mussel consumption should be limited to protect human health until more research can be conducted. It is therefore recommended that adults and children avoid wild mussel consumption to minimise non-carcinogenic and carcinogenic health risks from PCBs and OCPs. Local, farmed mussels should be investigated further to confirm if they can provide a lower-risk seafood option to consumers. It is evident that South African marine ecosystems are contaminated with a wide array of POPs, despite the country being signatories to the Stockholm Convention on POPs, and consistent monitoring of these ecosystems is essential to prevent further decline.

## Figures and Tables

**Table 1:** The co-ordinates, total number of mussels analysed, number of repeats, and species present per sampling location (with abbreviations) along the Western Cape Province coastline of South Africa in 2019.

Location	Abbrev.	Co-Ordinates		Reps ᶧ	Mussel #ᶧ	Mussel Species *		
						*MG*	*CM*	*PP*
Lambert’s Bay	LB	32°5′41.77” S	18°18′1.67” E	3	12	0	12	0
Eland’s Bay	EB	32°20′12.11” S	18°18′54.29” E	3	12	11	1	0
Saldanha Bay	SB	33°0′2.19” S	17°57′19.15” E	3	15	15	0	0
Mouille Point	MPL	33°54′0.85” S	18°24′5.55” E	3	15	1	14	0
Kommetjie	KL	34°8′50.24” S	18°19′5.85” E	3	12	11	1	0
Gordon’s Bay	GB	34°9′38.99” S	18°51′57.52” E	3	12	0	12	0
Hermanus	HM	34°25′16.54” S	19°14′38.85” E	3	12	11	1	0
Witsand	WS	34°23′46.38” S	20°51′28.80” E	3	12	0	0	12
Mossel Bay	MB	34°4′9.24” S	22°11′49.87” E	3	9	0	0	9
				Total	111	49	41	21

* Please note: *Mytilus galloprovincialis* = MG; *Choromytilus meridionalis* = CM; *Perna perna* = PP. ᶧ For Repeats (Reps) and Mussel #: The Mussel number is the total number of mussels analysed per location, while the Reps show how many repeats the total mussels were divided into (e.g., in Lambert’s Bay, 12 mussels were divided into three repeats, or 4 mussels per repeat sample).

**Table 2:** Minimum, maximum, and mean (ng g^−1^ dry weight ± SE) organic pollutant concentrations in wild mussels from the coastline of the Western Cape Province, South Africa (2019).

Class	Analyte	Full Analyte Name	Min *	Max	Mean ± Std Error
PAHs	Acenaphthene		3.20	11.43	6.9 ± 0.40
	Acenaphthylene		1.51	16.74	7.5 ± 0.68
	Anthracene		3.91	14.10	7.9 ± 0.43
	Benz(a)anthracene		NF	9.20	1.1 ± 0.36
	Benzo(ghi)perylene		NF	44.76	5.8 ± 1.91
	Chrysene		1.27	9.86	2.3 ± 0.36
	Fluorene		18.80	50.33	33.3 ± 1.45
	Fluoranthene		7.35	22.27	16.4 ± 0.68
	Naphthalene		2.77	9.30	4.5 ± 0.24
	Phenanthrene		8.16	21.57	14.1 ± 0.79
	Pyrene		7.58	27.19	17.7 ± 1.08
	ΣPAHs				117.8
PCBs	PCB 8	2,4′-Dichlorobiphenyl	7.02	9.14	7.5 ± 0.09
	PCB 18	2,2′,5-Trichlorobiphenyl	8.25	11.92	9.2 ± 0.17
	PCB 28	2,4,4′-Trichlorobiphenyl	4.92	18.55	6.4 ± 0.50
	PCB 44	2,2′,3,5′-Tetrachlorobiphenyl	6.04	8.67	6.6 ± 0.10
	PCB 52	2,2′,5,5′-Tetrachlorobiphenyl	4.12	6.51	4.5 ± 0.09
	ΣPCBs				34.4
OCPs	β-HCH	Beta-hexachlorocyclohexane	NF	7.95	5.2 ± 0.25
	Chlordane		7233	14,491	10,559 ± 344
	ΣOCPs				10,564.8

* NF stands for not found.

**Table 3:** A summary of the Estimated Daily Intakes, Hazard Quotient, and Total Hazard Index of low-, medium-, and high-level child and adult mussel consumers (250, 500, and 1000 g m^−1^, respectively) for mean and maximum concentrations of six PAHs detected in wild mussels from the Western Cape Province coastline of South Africa.

PAH	Site ^2^	Ci	RfDs ^1^	ADULT			CHILD		
				Low	Med	High	Low	Med	High
		ng g^−1^ w.w.	mg kg^−1^ bw day^−1^	Estimated Daily Intake (mg kg^−1^ bw day^−1^)					
Acenaphthene	WC	1.30	0.06	0.00015	0.00033	0.00066	0.00072	0.0015	0.0031
	GB	2.58	0.06	0.00031	0.00066	0.0013	0.0014	0.0031	0.0062
Anthracene	WC	1.48	0.3	0.00018	0.00038	0.00076	0.00082	0.0018	0.0035
	GB	3.08	0.3	0.00037	0.00078	0.0016	0.0017	0.0037	0.0073
Fluoranthene	WC	3.01	0.04	0.00036	0.00077	0.0015	0.0017	0.0036	0.0072
	GB	5.24	0.04	0.00062	0.0013	0.0027	0.0029	0.0062	0.0125
Fluorene	WC	6.25	0.04	0.00074	0.0016	0.0032	0.0035	0.0074	0.015
	GB	13.07	0.04	0.0016	0.0033	0.0067	0.0073	0.016	0.031
Naphthalene	WC	0.85	0.02	0.00010	0.00022	0.00043	0.00047	0.0010	0.0020
	HM	1.98	0.02	0.00024	0.00051	0.0010	0.0011	0.0024	0.0047
Pyrene	WC	3.21	0.03	0.00038	0.00082	0.0016	0.0018	0.0038	0.0077
	GB	6.00	0.03	0.00071	0.0015	0.0031	0.0033	0.0071	0.014
				Hazard Quotient * (Index Value)					
Acenaphthene	WC	1.30	0.06	0.0026	0.0055	0.011	0.012	0.026	0.051
	GB	2.58	0.06	0.0051	0.011	0.022	0.024	0.051	0.10
Anthracene	WC	1.48	0.3	0.0006	0.0013	0.0025	0.0027	0.0059	0.012
	GB	3.08	0.3	0.0012	0.0026	0.0052	0.0057	0.012	0.02
Fluoranthene	WC	3.01	0.04	0.0089	0.019	0.038	0.042	0.089	0.18
	GB	5.24	0.04	0.016	0.033	0.067	0.073	0.16	0.31
Fluorene	WC	6.25	0.04	0.019	0.040	0.080	0.087	0.19	0.37
	GB	13.07	0.04	0.039	0.083	0.17	0.18	0.39	0.78
Naphthalene	WC	0.85	0.02	0.005	0.011	0.022	0.024	0.050	0.10
	HM	1.98	0.02	0.012	0.025	0.051	0.055	0.12	0.24
Pyrene	WC	3.21	0.03	0.013	0.027	0.055	0.059	0.13	0.26
	GB	6.00	0.03	0.024	0.051	0.10	0.11	0.24	0.48
ΣHQs	WC		THI	0.048	0.10	0.21	0.23	0.48	0.97
	Max		THI	0.10	0.21	0.41	0.45	0.97	1.93

* Hazard Quotient values highlighted in red exceed the threshold for human health risks (>1). ^1^ All RfDs obtained from USEPA (2022) values provided in the Methodology Section. ^2^ WC stands for mean concentrations detected from the whole coast; GB represents samples from Gordon’s Bay; HM represents samples from Hermanus. These locations were where maximum concentrations of the selected PAH were determined.

**Table 4:** A summary of the Estimated Daily Intakes, Hazard Quotient, and Total Hazard Index of low-, medium-, and high-level child and adult mussel consumers (250, 500, and 1000 g m^−1^) for mean and maximum concentrations of the five PCBs detected in wild mussels from the Western Cape Province coastline of South Africa.

Contaminant	Location	Ci	ADULT			CHILD		
			Low	Medium	High	Low	Medium	High
		ng g^−1^ w.w.	Estimated Daily Intake (mg kg^−1^ bw day^−1^)					
PCB 8	WC coast	1.40	0.00017	0.00036	0.00071	0.00078	0.0017	0.0033
	GB ^1^	1.88	0.00022	0.00048	0.00096	0.0010	0.0022	0.0045
PCB 18	WC coast	1.70	0.00020	0.00043	0.00087	0.00094	0.0020	0.0040
	HM	2.55	0.00030	0.00065	0.0013	0.0014	0.0030	0.0061
PCB 28	WC coast	1.19	0.00014	0.00030	0.00061	0.00066	0.0014	0.0028
	GB	3.75	0.00045	0.00096	0.0019	0.0021	0.0045	0.0089
PCB 44	WC coast	1.22	0.00014	0.00031	0.00062	0.00068	0.0014	0.0029
	GB	1.69	0.00020	0.00043	0.00086	0.00094	0.0020	0.0040
PCB 52	WC coast	0.84	0.00010	0.00021	0.00043	0.00047	0.0010	0.0020
	GB	1.12	0.00013	0.00029	0.00057	0.00062	0.0013	0.0027
ΣPCBs	Mean	6.34	0.00075	0.0016	0.0032	0.0035	0.0075	0.015
	Max	11.00	0.0013	0.0028	0.0056	0.0061	0.013	0.026
			Hazard Quotient * (Index Value)					
PCB 8	WC coast	1.40	**2.37**	**5.09**	**10.17**	**11.07**	**23.74**	**47.47**
	GB	1.88	**3.20**	**6.86**	**13.72**	**14.94**	**32.03**	**64.04**
PCB 18	WC coast	1.70	**2.89**	**6.19**	**12.37**	**13.47**	**28.88**	**57.73**
	HM ^1^	2.55	**4.33**	**9.28**	**18.55**	**20.20**	**43.30**	**86.58**
PCB 28	WC coast	1.19	**2.02**	**4.33**	**8.65**	**9.42**	**20.20**	**40.38**
	GB	3.75	**6.38**	**13.68**	**27.36**	**29.78**	**63.85**	**127.67**
PCB 44	WC coast	1.22	**2.07**	**4.44**	**8.87**	**9.66**	**20.71**	**41.41**
	GB	1.69	**2.87**	**6.16**	**12.32**	**13.42**	**28.76**	**57.51**
PCB 52	WC coast	0.84	**1.43**	**3.07**	**6.14**	**6.68**	**14.33**	**28.65**
	GB	1.12	**1.91**	**4.09**	**8.18**	**8.91**	**19.10**	**38.18**
ΣHQs	Mean	Total Hazard Index	**10.78**	**23.11**	**46.21**	**50.30**	**107.85**	**215.65**
	Max	Total Hazard Index	**18.69**	**40.08**	**80.14**	**87.24**	**187.04**	**373.97**

* The Hazard Quotient was calculated using the RfD for Aroclor 1016: 7 E^−5^ mg kg^−1^ bw day^−1^. Values highlighted in red (bold) exceed the threshold for human health risks (>1). ^1^ GB represents samples from Gordon’s Bay and HM represents samples from Hermanus; these locations were where maximum concentrations of the selected PAH were determined.

**Table 5:** A summary of the potential cancer risks associated with low (250 g m^−1^), medium (500 g m^−1^) and high (1000 g m^−1^) consumption rates of wild mussels from the Western Cape Province coastline.

Contaminant	Location	Cancer Risk by Consumption Rate *					
		ADULT			CHILD		
		Low	Med	High	Low	Med	High
PCB 8	WC coast	**0.00033**	**0.00071**	**0.0014**	**0.0016**	**0.0033**	**0.0066**
	GB	**0.00045**	**0.0010**	**0.0019**	**0.0021**	**0.0045**	**0.0090**
PCB 18	WC coast	**0.00040**	**0.0009**	**0.0017**	**0.0019**	**0.0040**	**0.0081**
	HM	**0.00061**	**0.0013**	**0.0026**	**0.0028**	**0.0061**	**0.012**
PCB 28	WC coast	**0.00028**	**0.00061**	**0.0012**	**0.0013**	**0.0028**	**0.0057**
	GB	**0.00089**	**0.0019**	**0.0038**	**0.0042**	**0.0089**	**0.018**
PCB 44	WC coast	**0.00029**	**0.00062**	**0.0012**	**0.0014**	**0.0029**	**0.0058**
	GB	**0.00040**	**0.00086**	**0.0017**	**0.0019**	**0.0040**	**0.0081**
PCB 52	WC coast	**0.00020**	**0.00043**	**0.00086**	**0.00094**	**0.0020**	**0.0040**
	GB	**0.00027**	**0.00057**	**0.0011**	**0.0012**	**0.0027**	**0.0053**
ΣPCBs	Mean	**0.0015**	**0.0032**	**0.0065**	**0.0070**	**0.015**	**0.030**
	Max	**0.0026**	**0.0056**	**0.011**	**0.012**	**0.026**	**0.052**

* Values in red (bold) have an unacceptable cancer risk > 10^−4^.

**Table 6:** Summary of the child and adult Estimated Daily Intakes (EDIs), Hazard Quotients, and cancer risks for low (250 g m^−1^), medium (500 g m^−1^), and high (1000 g m^−1^) consumption levels of wild mussels contaminated by chlordane.

Group	Location	Consumption Rate	Chlordane Conc.	EDI	Hazard Quotient ¹	Cancer Risk *
			mg kg^−1^ w.w.	mg kg^−1^ bw day^−1^	Index Value	Index Value
Adult	WC coast	Low	1.95	0.23	**462.98**	**0.081**
	(mean)	Medium		0.50	**992.66**	**0.17**
		High		0.99	**1984.76**	**0.35**
	GB (max)	Low	3.42	0.41	**815.07**	**0.14**
		Medium		0.87	**1747.56**	**0.31**
		High		1.75	**3494.14**	**0.61**
Child	WC coast	Low	1.95	1.08	**2160.58**	**0.38**
	(mean)	Medium		2.32	**4632.40**	**0.81**
		High		4.63	**9262.20**	**1.62**
	GB (max)	Low	3.42	1.90	**3803.66**	**0.67**
		Medium		4.08	**8155.28**	**1.43**
		High		8.15	**16** **,** **305.99**	**2.85**

* Values in red text (bold) have an unacceptable cancer risk (>10^−4^). ^1^ Values highlighted in red exceed the threshold for risk (>1).

**Table 7:** A summary of the child and adult Estimated Daily Intakes (EDIs), Hazard Quotients, and cancer risks for low- (250 g m^−1^), medium- (500 g m^−1^), and high-level (1000 g m^−1^) mussel consumers of wild mussels contaminated with β-HCH.

**Age Group**	**Location**	**Consumption Rate**	**β-HCH Conc.**	**EDI**	**Hazard Quotient ¹**	Cancer Risk *
			ng g^−1^ w.w.	mg kg^−1^ bw day^−1^	Index Value	Index Value
Adult	WC Coast	Low	0.98	0.00012	0.39	**0.00021**
	(mean)	Med		0.00025	0.83	**0.00045**
		High		0.00050	**1.66**	**0.00090**
	HM	Low	1.69	0.00020	0.67	**0.00036**
	(max)	Med		0.00043	**1.44**	**0.00078**
		High		0.00086	**2.88**	**0.0016**
Child	WC Coast	Low	0.97	0.0005	**1.81**	**0.0010**
	(mean)	Med		0.0012	**3.87**	**0.0021**
		High		0.0023	**7.74**	**0.0042**
	HM	Low	1.69	0.00094	**3.14**	**0.0017**
	(max)	Med		0.0020	**6.73**	**0.0036**
		High		0.0040	**13.45**	**0.0073**

* Values in black text fall into an “area of concern” regarding cancer risks (between 10^−4^ and 10^−6^), and values in red text (bold) have an unacceptable cancer risk (>10^−4^). ^1^ Values highlighted in red exceed the threshold for risk (>1).

## Data Availability

The original contributions presented in this study are included in the article/supplementary material. Further inquiries can be directed to the corresponding authors.

## Supplementary Materials

The following supporting information can be downloaded at https://www.mdpi.com/article/10.3390/foods14132226/s1, Figure S1: PCB concentrations (ng g^−1^ d.w.) in wild mussels from the coastline of the Western Cape Province, South Africa; Figure S2: Concentrations of β-HCH (ng g^−1^ d.w.) in wild mussels from the coastline of the Western Cape Province, South Africa; Figure S3: Concentrations of chlordane (ng g^−1^ d.w.) in wild mussels from the coastline of the Western Cape Province, South Africa.

## Abbreviations

The following abbreviations are used in this manuscript:

CECs	Contaminants of Emerging Concern
DAFF	Department of Agriculture, Forestry, and Fisheries
DDT	Dichlorodiphenyltrichloroethane
d.w.	Dry Weight
EC	European Commission
EDI	Estimated Daily Intake
ePOPs	Emergent POPs
HCH	Hexachlorocyclohexane
HHRA	Human Health Risk Assessment
HMW	High Molecular Weight
HQ	Hazard Quotient
LMW	Low Molecular Weight
NDL-PCBs	Non-Dioxin-Like PCBs
NF	Not Found
OCPs	Organochlorine Pesticides
OPPs	Organophosphate Pesticides
PAHs	Polyaromatic Hydrocarbons
PBDEs	Polybrominated Diphenyl Ethers
PCBs	Polychlorinated Biphenyls
PCDDs	Polychlorinated Dibenzo-p-dioxins (also known as dioxins)
PCDFs	Polychlorinated Dibenzofurans (also known as furans)
POPs	Persistent Organic Pollutants
RfD	Reference Dose
SAMSM&CP	South African Molluscan Shellfish Monitoring and Control Programme
THI	Total Hazard Index
UNEP	United Nations Environmental Programme
USEPA	United States Environmental Protection Agency
w.w.	Wet Weight
MDPI	Multidisciplinary Digital Publishing Institute
