# Supplementary material for: A Human Health Risk Assessment of Persistent Organic Pollutants in Wild Marine Mussels from the Western Cape Province of South Africa
Source: Foods. 2025 Jun 24;14(13):2226. doi: 10.3390/foods14132226 (PMC12249414; doi:10.3390/foods14132226)
Supplement: Supplementary file 1 [file foods-14-02226-s001.zip › foods-3688459-supplementary.pdf]

Supplementary Materials

S1. Polychlorinated Biphenyls (PCBs)

Five PCB congeners were detected in wild mussels from the Western Cape Province coastline; these were the PCBs 8, 18, 28, 44 and 52. As shown in Figure S1, the PCB with the highest concentration was PCB 18 (min: 8.8 ng g^{-1} d.w. in Kommetjie, max: 9.8 ng g^{-1} d.w. in Gordon's Bay), followed by PCB 8 (min: 7.3 ng g^{-1} d.w. in Hermanus, max: 8.0 ng g^{-1} d.w. in Lambert's Bay), PCB 28 (min: 5.4 ng g^{-1} d.w. in Mossel Bay, max: 9.8 ng g^{-1} d.w. in Gordon's Bay), PCB 44 (min: 6.4 ng g^{-1} d.w. in Hermanus & Mossel Bay, max: 7.3 ng g^{-1} d.w. in Lambert's Bay), with the lowest concentrations found for PCB 52 (min: 4.3 ng g^{-1} d.w. in Hermanus, max: 5.3 ng g^{-1} d.w. in Lambert's Bay). Most PCBs had very consistent concentrations in wild mussels, and while PCB 28 showed fluctuations between differing sampling locations (with peaks in Lambert's Bay and Gordon's Bay mussels); these differences were not found to be statistically significant ($p \geq 0.05$).

S2. Organochlorine Pesticides (OCPs)

Only two OCPs were detected in wild mussels from the Western Cape Province coastline; β-HCH and chlordane. The concentrations of β-HCH from nine different sampling sites is given in Figure S2; β-HCH was highest in samples from Hermanus (6.5 ng g^{-1} d.w.) and lowest in samples from Witsand (3.9 ng g^{-1} d.w.). The high variation in the standard error of the mean for samples from Witsand was due to β-HCH being undetected (or Not Found) within one of the three repeats. In Figure S3 it is shown that chlordane concentrations were highest in samples from Mouille Point (11 767 ng g^{-1} d.w.) and lowest in Saldanha Bay (9 240 ng g^{-1} d.w.). Statistical analysis found that there were no significant differences in β-HCH and chlordane concentrations between locations ($p = 0.63$ and $p = 0.55$, respectively).

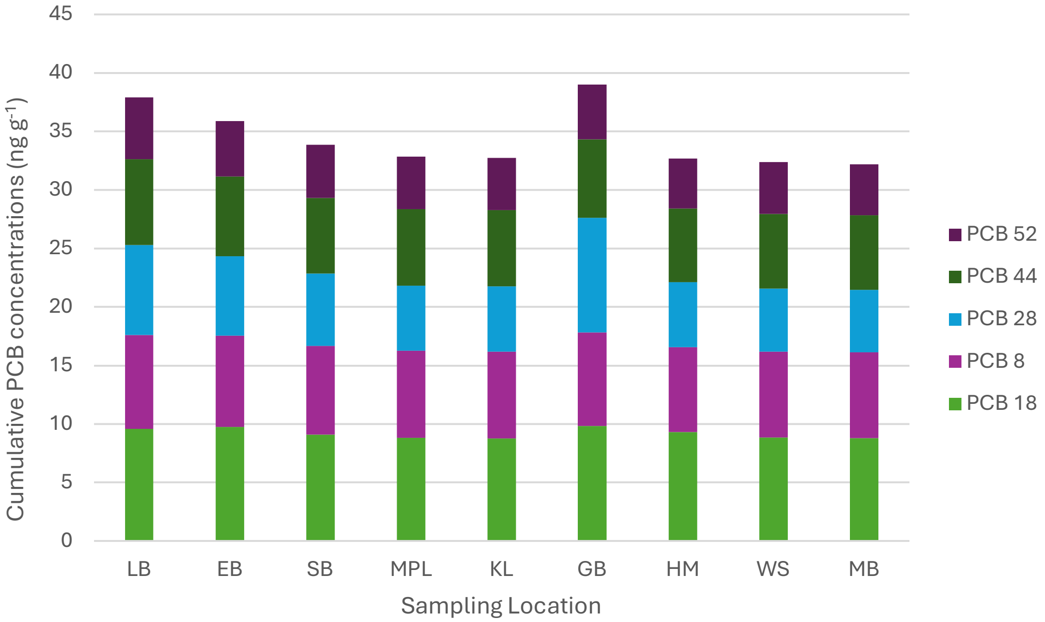

Figure S1. PCB concentrations (ng g^{-1} d.w.) in wild mussels from the coastline of the Western Cape Province, South Africa.

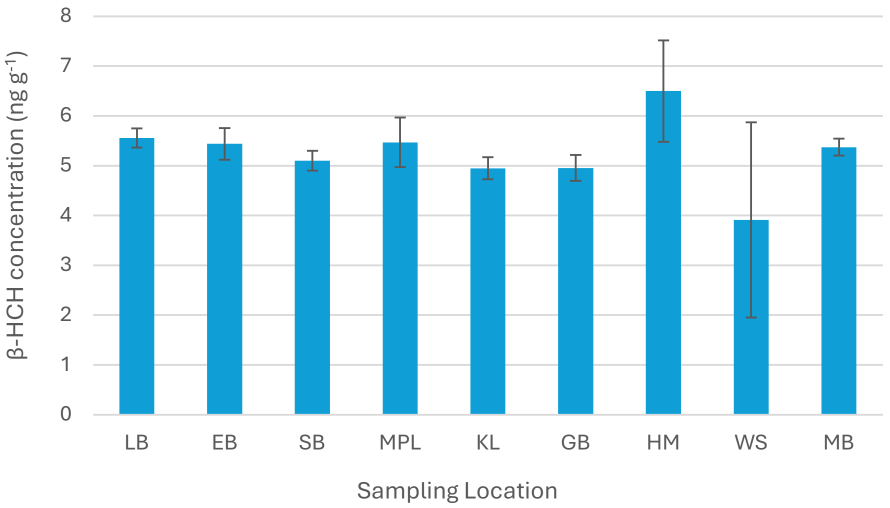

Figure S2. Concentrations of β-HCH (ng g^{-1} d.w.) in wild mussels from the coastline of the Western Cape Province, South Africa.

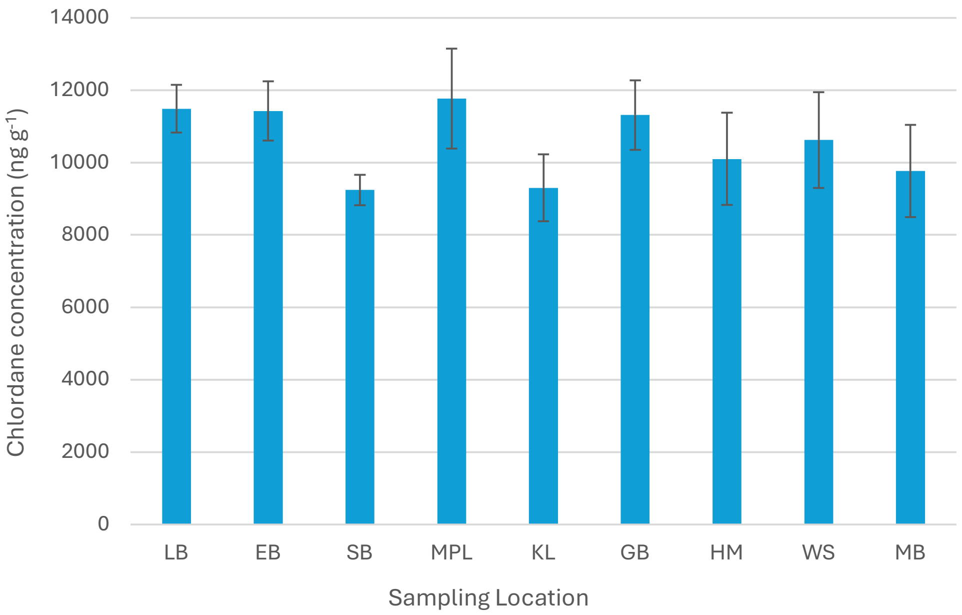

Figure S3. Concentrations of chlordane (ng g^{-1} d.w.) in wild mussels from the coastline of the Western Cape Province, South Africa.
